# Cysteine proteinase cathepsin H in tumours and sera of lung cancer patients: relation to prognosis and cigarette smoking

**DOI:** 10.1054/bjoc.1999.0999

**Published:** 2000-02

**Authors:** A Schweiger, A Staib, B Werle, M Kras̆ovec, T T Lah, W Ebert, V Turk, J Kos

**Affiliations:** Department of Biochemistry and Molecular Biology, Joz̆ef Stefan Institute, Jamova 39, Ljubljana, 1000, Slovenia; Department of Clinical Chemistry and Bacteriology, Thoraxhospital Heidelberg-Rohrbach, D-69126, Germany; Department of Biochemical Research and Drug Design, Krka d.d.R&D Division, Ljubljana, 1000, Slovenia; National Institute of Biology, Ljubljana, 1000, Slovenia

**Keywords:** cathepsin H, tumour markers, survival, ELISA, cigarette smoke

## Abstract

In order to evaluate the role of cysteine peptidase cathepsin H (Cath H) in human lung cancer its protein levels were determined in 148 pairs of lung tumour tissue and adjacent non-tumourous lung parenchyma using the enzyme-linked immunosorbent assay technique. Additionally, Cath H levels were determined in sera of 171 patients with malignant tumours, 34 patients with benign lung diseases and 47 healthy controls. The median level of Cath H in tumour tissue was 0.64 times that in the corresponding lung parenchyma. Relating tumour levels with histological type we found higher Cath H levels in small-cell and adenocarcinomas and lower levels in squamous cell carcinoma, large-cell carcinoma and secondary tumours. A significant difference in Cath H level between lung tumour tissue and non-tumourous lung parenchyma was associated with the group of cigarette smokers (156 vs 263 ng mg^−1^protein, *P*< 0.001). For this group of patients Cath H tumour levels correlated with the survival rate, while for the entire patient population this was not the case. Smokers with high tumour levels of Cath H experienced poor survival. Cath H was significantly higher in sera of patients with malignant and benign lung diseases than in control sera (*P*< 0.001). The increase was significant for all histological types, being the highest in small-cell and squamous cell carcinomas. Our study reveals that in lung tumours there is different behaviour of Cath H compared with other cysteine peptidases, e.g. cathepsin B and cathepsin L. Variations between tissue and serum levels of Cath H indicate either reduced expression or enhanced secretion of this enzyme in lung tumours. © 2000 Cancer Research Campaign


*British Journal of Cancer* (2000) **82**(4), 782–788
© 2000 Cancer Research Campaign
DOI: 10.1054/ bjoc.1999.0999, available online at http://www.idealibrary.com on IDEAL®

### Cysteine proteinase cathepsin H in tumours and sera of lung cancer patients: relation to prognosis and cigarette smoking

**A Schweiger[1], A Staib[2], B Werle[2], M Krašovec[3], TT Lah[4], W Ebert[2], V Turk[1] and J Kos[1,3]**

[1]Jožef Stefan Institute, Department of Biochemistry and Molecular Biology, Jamova 39, 1000 Ljubljana, Slovenia; [2]Thoraxhospital Heidelberg-Rohrbach, Department of Clinical Chemistry and Bacteriology, D-69126 Germany; [3]Krka, d.d., R&D Division, Department of Biochemical Research and Drug Design, 1000 Ljubljana, Slovenia; [4]National Institute of Biology, 1000 Ljubljana, Slovenia.

**Summary** In order to evaluate the role of cysteine peptidase cathepsin H (Cath H) in human lung cancer its protein levels were determined in 148 pairs of lung tumour tissue and adjacent non-tumourous lung parenchyma using the enzyme-linked immunosorbent assay technique. Additionally, Cath H levels were determined in sera of 171 patients with malignant tumours, 34 patients with benign lung diseases and 47 healthy controls. The median level of Cath H in tumour tissue was 0.64 times that in the corresponding lung parenchyma. Relating tumour levels with histological type we found higher Cath H levels in small-cell and adenocarcinomas and lower levels in squamous cell carcinoma, large-cell carcinoma and secondary tumours. A significant difference in Cath H level between lung tumour tissue and non-tumourous lung parenchyma was associated with the group of cigarette smokers (156 vs 263 ng mg$^{-1}$ protein, $P < 0.001$). For this group of patients Cath H tumour levels correlated with the survival rate, while for the entire patient population this was not the case. Smokers with high tumour levels of Cath H experienced poor survival. Cath H was significantly higher in sera of patients with malignant and benign lung diseases than in control sera ($P < 0.001$). The increase was significant for all histological types, being the highest in small-cell and squamous cell carcinomas. Our study reveals that in lung tumours there is different behaviour of Cath H compared with other cysteine peptidases, e.g. cathepsin B and cathepsin L. Variations between tissue and serum levels of Cath H indicate either reduced expression or enhanced secretion of this enzyme in lung tumours. © 2000 Cancer Research Campaign

**Keywords:** cathepsin H; tumour markers; survival; ELISA; cigarette smoke

Irregular functioning of cysteine proteinases cathepsin (Cath) B, H and L has been proposed as being involved in the development of various diseases, including cancer (Sloane et al, 1994; Schmitt et al, 1995; Duffy et al, 1996; Kos et al, 1998). Numerous studies have demonstrated a correlation of increased proteolytic activity of cysteine cathepsins with neoplastic transformation, tumour invasion and metastasis. Furthermore, the levels of these enzymes in tumours and some extracellular fluids have been shown to allow the disease-free and overall survival period to be predicted and therefore may serve as prognostic factors for cancer patients. However, the investigations were mainly focused on Cath B and Cath L, whereas the role of Cath H in malignant progression was much less studied and remains contradictory.

Increased levels of Cath H have been observed in glioblastoma and anaplastic astrocytoma when compared with normal brain tissue and low-grade gliomas (Sivaparvathi et al, 1996). Higher levels were also found in tumour tissue and sera of patients with breast cancer (Gabrijelčič et al, 1992) and in sera of melanoma patients (Kos et al, 1997). However, in head and neck tumours the levels of Cath H were lower in tumour tissue when compared to adjacent control tissue (Kos et al, 1995). Moreover, the lower levels of Cath H were associated with shorter disease-free survival period (Budihna et al, 1996). In the same study the opposite was true for Cath B and Cath L.

Cath H has also been shown to be a prognostic marker in melanoma patients. Patients with high serum levels of Cath H experienced significantly shorter overall survival than patients with low levels of the enzyme (Kos et al, 1997). In addition it was shown to predict the effectiveness of chemoimmunotherapy exhibiting significantly higher levels in non-responders than in responders (Kos et al, 1997).

In lung carcinoma the behaviour of Cath H has not so far been studied, although increased activity and/or concentrations of Cath B (Treftz et al, 1989; Krepela et al, 1990; Ebert et al, 1994; Knoch et al, 1994; Ledakis et al, 1996, Werle et al, 1997*a*), Cath L (Ledakis et al, 1996; Werle et al, 1997*a*) and Cath S (Werle et al, 1999b) are well documented in lung tumour cytosols and tissue sections. Higher levels of Cath B activity (Ebert et al, 1994) and of Cath L protein (Werle et al, 1997a) were found to correlate with shorter survival of patients with lung carcinoma.

The aim of the present study was to determine the levels of Cath H in lung tumours and their surrounding histologically non-cancerous lung parenchyma. Additionally, the levels of Cath H were determined in sera of patients with histologically distinct lung tumours and compared to controls. Results have been related to histopathological and clinical features, considering especially the correlation of individual tumour levels of Cath H with survival and with the smoking history of patients.

*Received 20 April 1999*
*Revised 6 September 1999*
*Accepted 14 September 1999*

*Correspondence to:* J Kos, Jožef Stefan Institute, Department of Biochemistry and Molecular Biology, Jamova 39, SI 1000 Ljubljana, Slovenia

### MATERIALS AND METHODS

### Patients

Lung tumour tissue and adjacent control (non-cancerous) lung parenchyma were obtained as matched paired samples from 148 lung cancer patients treated by surgery at the Thoraxhospital Heidelberg-Rohrbach. The age of patients in this group ranged from 15 to 78 (median 60). A major proportion were smokers ($n$ = 90, 60.8%), 17 (11.5%) were ex-smokers, having stopped smoking at least 0.25 years before surgery, 41 (27.7%) were non-smokers, or stopped smoking at least 5 years before surgery. The cell type of lung cancer was classified according to the WHO protocol and was based on predominant cell type (World Health Organization, 1981). The tumour disease stage (pTNM) was classified according to the international staging system (Hermanek and Sobin, 1987).

The second group of patients consisted of 171 patients with malignant tumours and 34 patients with benign diseases included in an ongoing prospective study on prognostic values of extracellular cysteine peptidases and their inhibitors in lung cancer. From patients' malignant tumours only Cath H post-therapy serum levels were included in the study. The median age and the range for these patients is comparable to the previous group. Out of 171 patients with malignant tumours 60 were small-cell, 67 adeno and 44 squamous cell type. The group of patients with benign diseases consisted of patients with pulmonary embolism, fibrosis, bronchostenosis, sarcoidosis, pneumonia, tuberculosis, pleuritis/emphysema, congestive heart failure, actinomycosis, bronchiectases and bronchial asthma. As a control group, sera from 47 healthy blood donors were collected (Kos et al, 1998).

### Sample collection

Tissue homogenization was carried out as described by Werle et al (1995). Five-millilitre blood samples were collected from lung cancer patients after therapy. The blood was clotted at 4–8°C and centrifuged at 3000 rpm. The sera were stored at –30°C until analysed. Sera from blood donors were sampled in a similar way.

### Determination of cathepsin H

Cath H concentrations were determined by enzyme-linked immunosorbent assay (ELISA) (sandwich ELISA; Krka d.d., Novo mesto, Slovenia) developed at Jožef Stefan Institute (Ljubljana, Slovenia). The components were purified and characterized, and the test optimized as described (Schweiger et al, 1997). The sheep polyclonal and murine 1D10 monoclonal antibodies recognize precursor and mature forms of Cath H, as well as enzyme–inhibitor complexes (Schweiger et al, 1997).

Linearity of the ELISA was tested by serial dilution of tissue cytosols or sera to levels within the range of the assay (Schweiger et al, 1997). The measured values of diluted samples were subsequently compared with standard values. Recovery was tested by the addition of different amounts of antigen to samples with known antigen concentration and was found to vary between 87.4 and 104.9%. A microplate reader (SLT Rainbow, Austria) was used to measure absorbance in ELISA. Cath H was expressed in ng $mg^{-1}$ of total protein. The detection limit of the assay was 2 ng $ml^{-1}$.

Tissue samples at 1:50 dilution and serum samples at 1:2 dilution were added to wells of a microtitre plate previously precoated with sheep polyclonal antibody to Cath H. The assay was then performed as described (Schweiger et al, 1997). After 2 h of incubation at 37°C the wells were washed and murine 1D10 monoclonal antibody, purified by affinity chromatography on Protein A Sepharose and conjugated subsequently with horseradish peroxidase (HRP) was added. After a further 2 h incubation at 37°C, peroxidase substrate 3,3,5,5-tetramethyl benzidine (TMB, Sigma, St Louis, MO, USA) in the presence of hydrogen peroxide was added. The amount of degraded substrate, as a measure of bound immuno-complexed Cath H, was measured by absorbance at 450 nm and the Cath H concentration was calculated from the calibration curve.

### Protein determination

Protein concentrations were determined according to Bradford et al (1976). Bovine serum albumin was used as standard.

### Statistical analysis

The results of Cath H measurements in the group under study are given as 5%, 50% (median) and 95% percentiles. For comparing the data of matched pairs of tumour and lung tissue, Wilcoxon's rank test was used. Differences in Cath H levels (tissue and serum) between various groups of patients were tested by Mann–Whitney and Kruskal–Wallis test. Univariate analysis of survival probability was performed by Kaplan–Meier analysis (Kaplan and Meier, 1958), using the log-rank test for determining statistical significance between survival curves. Critlevel program (Abel et al, 1984) was used for dichotomization of variables into low and high groups. In all tests, two-sided $P$-values below 5% were considered significant. Various statistical packages (SPSS program, SPSS Inc., Illinois, USA; PC-Statistic, TOPSOFT, Hannover, Germany; Statistica, StatSoft, Hamburg, Germany) were used.

### RESULTS

### Distribution of Cath H in lung tumours and in non-tumourous lung parenchyma

The concentrations of Cath H in 148 tissue homogenates of lung tumours and the corresponding non-tumourous lung parenchyma are summarized in Table 1. In the total patient population the median of Cath H concentration in tumour tissue was 0.64 times that in the control lung parenchyma.

The data were further analysed with respect to tumour histology, the anatomical spread out of the tumour (pTNM-stages), the degree of cell differentiation and smoking habits (Table 1). Regarding tumour histology, as seen in Figure 1, we found the lowest median value of Cath H in tumour tissues, compared to their control lung counterparts, in large-cell carcinomas and in squamous cell carcinomas. The ratios for both carcinomas were significantly different ($P$ = 0.001 and $P$ = 0.028 respectively). In contrast, in carcinoids ($n$ = 5) the median level of Cath H was 1.7-fold higher in tumour tissue than in lung parenchyma, but the difference was not statistically significant.

The highest median value of Cath H was detected in small-cell carcinomas (SCLC) and in adenocarcinomas (AC). While low numbers of SCLC ($n$ = 3) did not permit reliable statistical

© 2000 Cancer Research Campaign

**Table 1** Cathepsin H in lung tumour homogenates

| | *n* | Cathepsin H concentration (ng mg$^{-1}$ protein) Tumour median (5%, 95%) | Lung median (5%, 95%) | Tu/Lu median | *P* |
|---|---|---|---|---|---|
| Total | 148 | 161 (45, 864) | 253 (33, 767) | 0.64 | NS |
| SCC | 58 | 141 (46, 824) | 255 (44, 824) | 0.55 | 0.001 |
| AC | 56 | 221 (51, 1404) | 264 (25, 836) | 0.84 | NS |
| LC | 6 | 93 (72, 407) | 325 (209, 528) | 0.29 | 0.028 |
| Carcinoid | 5 | 134 (23, 690) | 78 (25, 396) | 1.7 | NS |
| Others | 1 | 102 (102, 102) | 207 (207, 207) | 0.49 | ND |
| SCLC | 3 | 233 (43, 398) | 287 (62, 635) | 0.81 | NS |
| Secondary tumours | 19 | 126 (18, 685) | 150 (69, 1344) | 0.84 | NS |
| pT1 | 19 | 156 (47, 3306) | 339 (17, 2266) | | NS |
| pT2 | 79 | 155 (44, 820) | 211[a] (30, 685) | | NS |
| pT3 | 20 | 162 (45, 1496) | 317 (15, 685) | | 0.014 |
| pT4 | 11 | 244 (61, 988) | 591 (89, 1061) | | 0.041 |
| pN0 | 43 | 173 (37, 971) | 259 (19, 871) | | NS |
| pN1 | 41 | 119 (44, 729) | 253 (47, 812) | | 0.001 |
| pN2 | 26 | 206 (65, 1772) | 335 (80, 694) | | NS |
| pN3 | 19 | 168 (53, 3306) | 287 (10, 1061) | | NS |
| G1/G2[b] | 41 | 191 (30, 1296) | 248 (27, 772) | | NS |
| G3 | 104 | 142 (46, 799) | 248 (46, 807) | | NS |
| pTNMI/II/IIIa | 93 | 157 (45, 844) | 259 (28, 697) | | 0.005 |
| pTNMIII/b/IV | 55 | 166 (46, 1054) | 226 (65, 927) | | 0.016 |
| Smokers | 90 | 156 (44, 1032) | 263 (33, 856) | | 0.001 |
| Ex-smokers | 17 | 164 (53, 901) | 260 (32, 781) | | 0.039 |
| Non-smokers | 41 | 173 (31, 683) | 181 (30, 740) | | NS |

NSCLC: non small cell lung carcinoma; SCC: squamous cell carcinoma; AC: adenocarcinoma; LC: large cell carcinoma; SCLC: small-cell lung cancer; Tu: tumour tissue; Lu: lung parenchyma; [a] Cath H concentrations in normal lung parenchyma were significantly lower in pT2 stage as compared to other stages ($P$ = 0.004); [b]Cell differentiation could not be assessed for three patients; NS (not significant); ND (not determined).

evaluation, Cath H was significantly higher in AC than in squamous cell carcinomas (SCC), in large-cell carcinomas (LCC) and in secondary tumours (i.e. metastases to the lung). There was no statistically significant correlation between Cath H levels and cell differentiation, lymph node involvement and pTNM-staging. However, in non-tumourous lung tissue significantly lower levels of Cath H were found in patients with tumour size pT2 compared to those with sizes pT1, pT3 and pT4 ($P$ = 0.006, $p$ = 0.035 and $P$ = 0.023 respectively).

Comparing Cath H levels in tumour tissue with those in lung parenchyma, significant differences in patients with tumour size pT3 and pT4 ($P$ = 0.014 and $P$ = 0.041 respectively), in patients with lymph node metastasis pN1 ($P$ = 0.001) and in patients with pTNM-stage I, II, IIIa and pTNM-stage IIIb, IV ($P$ = 0.005 and $P$ = 0.01 respectively) are seen.

With regard to smoking habits, Cath H levels of non-tumourous lung parenchyma were significantly higher in smokers than in non-smokers ($P$ = 0.042) and are shown in Figure 2. There were

 *© 2000 Cancer Research Campaign*

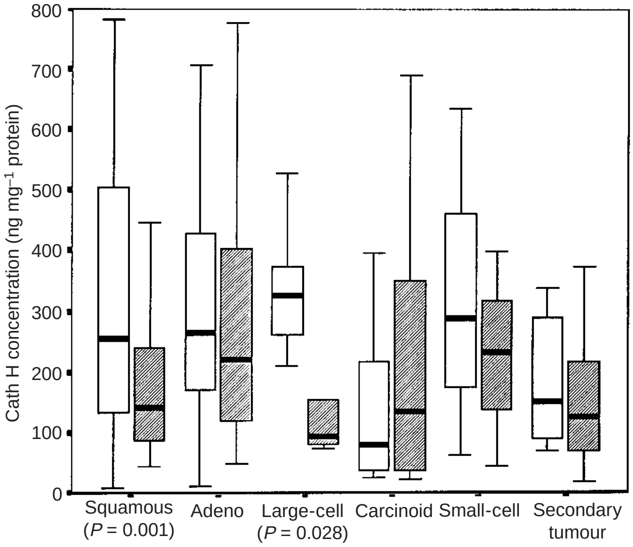

**Figure 1** Cath H levels in matched pairs of lung parenchyma (□) and tumour tissues (▨). The bold line in the box is the median value. The top and the bottom of the box represent 25th and 75th percentiles, respectively, and the ends of the bars represent the 5th and 95th percentiles

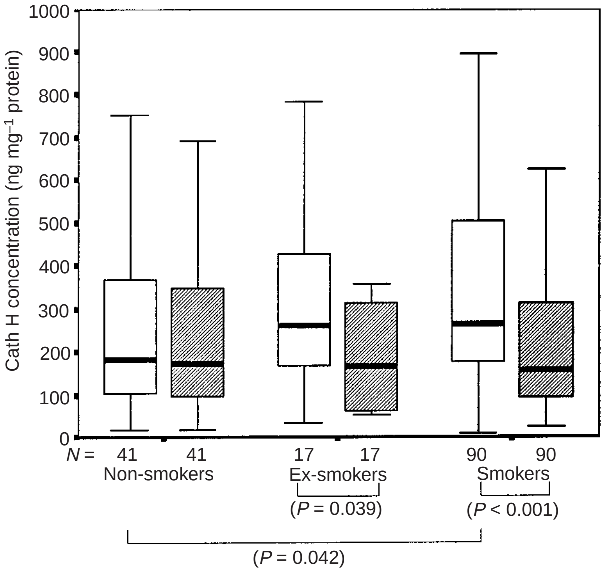

**Figure 2** Cath H levels in matched pairs of lung parenchyma (□) and tumour tissues (▨) of non-smokers, ex-smokers and smokers. The bold line in the box is the median value. *n* represents the number of patients in the sub-group. The top and the bottom of the box represent 25th and 75th percentiles, respectively, and the ends of the bars represent the 5th and 95th percentiles also significant differences in Cath H levels between non-tumourous lung parenchyma and lung tumour tissue in smokers ($P < 0.001$) and ex-smokers ($P = 0.039$), whereas for non-smokers no significant differences could be observed.

### Detection of Cath H in sera of patients with lung tumours

The levels of Cath H measured in 205 serum samples from patients suffering from lung diseases and from 47 healthy humans are listed in Figure 3. Compared to healthy controls, the level of Cath H was significantly increased in sera of lung cancer patients regardless of their histological type ($P < 0.001$). The highest levels were observed in SCLC and in SCC. The levels of both histological types were significantly higher than those from patients suffering from benign lung diseases ($P = 0.019$ and $P = 0.0003$ respectively).

### Survival analysis

Analysis of the entire patient population revealed no significant correlation between Cath H levels and overall survival probability. However, within the subgroup of smokers, Cath H levels correlated with survival probability, since 11 out of 18 patients with Cath H levels above 349 ng $mg^{-1}$ died in the observation period of 4.6 years compared to 29 out of 68 patients below that cut-off level (Figure 4). For the subgroup of non-smokers the correlation of Cath H with overall survival probability was not significant.

### DISCUSSION

In our study on 148 patients with lung carcinomas we found levels of Cath H concentration in tumour tissue 0.64 times those from adjacent lung parenchyma. A similar observation has been reported for head and neck carcinoma (Kos et al, 1995; Budihna et al, 1996) with 0.42 times lower Cath H levels in tumour tissue than in controls.

Relating tumour tissue levels of Cath H with histological cell type we found significantly higher Cath H levels in SCLC and AC than in SCC, LC and secondary tumours. There was no correlation of Cath H tumour level with tumour size, lymph node involvement, pTNM-stage and cell differentiation (grading).

Rather high levels of Cath H were found in non-tumourous lung parenchyma of patients with LC, whereas the lowest levels of Cath H were observed in pulmonary carcinoids. It should be noted that LC are high-grade neoplasms, whereas pulmonary carcinoids are low-grade neoplasms, with 5-year overall survival rates of about 11% and 70%, respectively (McCaughan et al, 1985; Mountain et al, 1991).

Separating patients into smokers and non-smokers, a significant difference between tumour and control Cath H levels appeared only within the former group, whereas for non-smokers Cath H remained unchanged. This is the first evidence indicating association of Cath H expression or regulation with cigarette smoking in human lung cancer and is consistent with previous studies revealing an increase of Cath B and L activity in lung tissue of smokers (Knoch et al, 1994; Werle et al, 1995), in their alveolar macrophages and alveolar lavage fluid (Chang et al, 1986; Takahashi et al, 1993) as well as in alveolar macrophages and bronchoalveolar lavage fluid of rats exposed to cigarette smoke (Gairola et al, 1989; Lesser et al, 1989; Chapman et al, 1997). Furthermore, Yukio Ishii (1991*a*, 1991*b*) clearly demonstrated that Cath H is secreted mainly with surfactant from Type II alveolar epithelial cells and alveolar macrophages of rat lung. This report strongly indicates a different role of Cath H from those of Cath B and Cath L. In fact, contrary to Cath H, the overexpression of Cath B and Cath L has been reported in lung tumours as well as in several other studies of aetiologically different cancers (reviewed by Kos et al, 1998). Although Cath B, L and H are all lysosomal cysteine peptidases, large variations in expression of individual cathepsins in different tissues (Qian et al, 1991) and even in

© 2000 Cancer Research Campaign

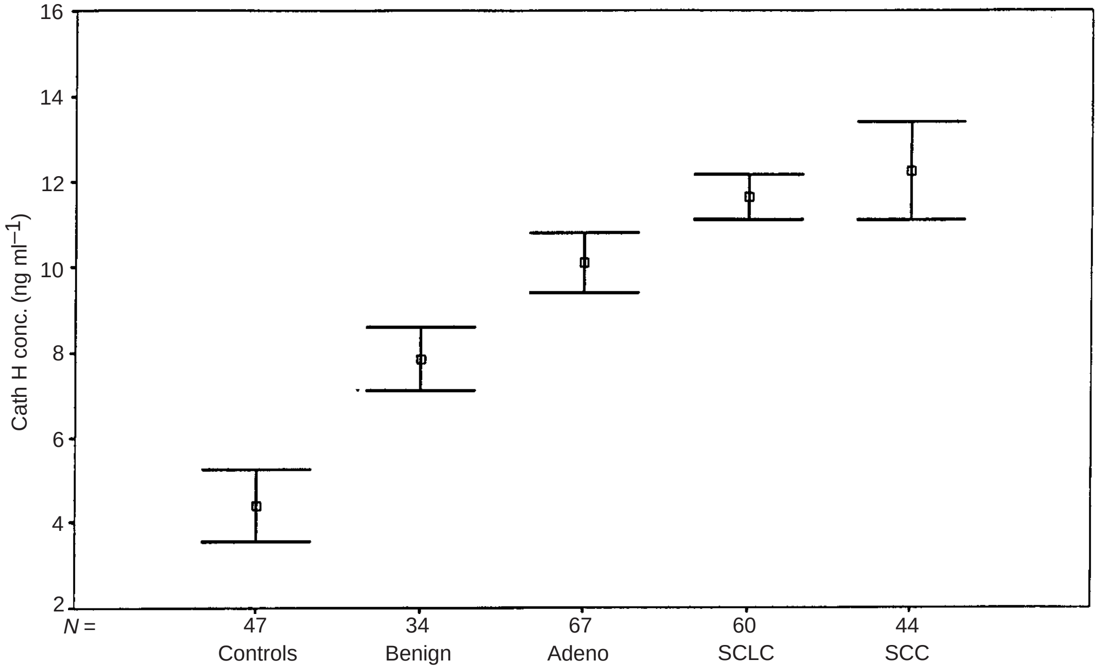

**Figure 3** Cath H levels in sera of patients with lung tumours and healthy controls. Error bars represent mean ± 2 s.e.m

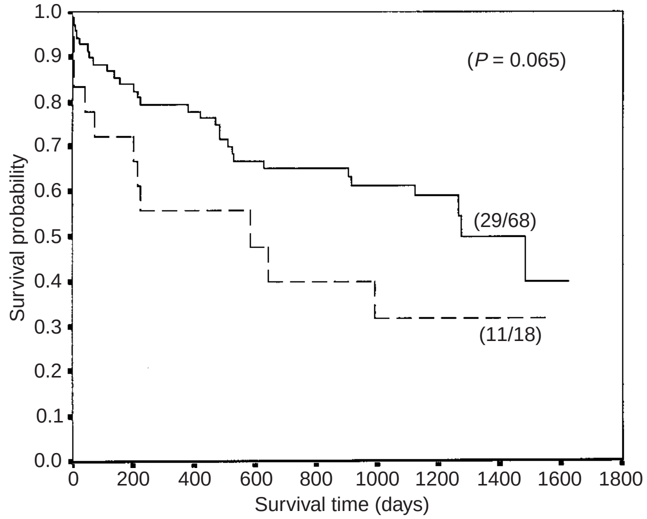

**Figure 4** Prognostic significance of Cath H within the group of smokers. A cut-off value of 349 ng mg$^{-1}$ protein to divide patients into low- (—— Cath H < 349 ng mg$^{-1}$ prot.) and high- -------- Cath H ≥ 349 ng mg$^{-1}$ prot.) groups different types of cells within the same tissue (Uchiyama et al, 1994) have been reported. Selective expression of individual peptidases in different types of tumour suggest that they may participate in specialized cellular functions (San Segundo et al, 1986; Chapman et al, 1997). Distinct function of these enzymes could result from the differences in their structure. In contrast to Cath L, which is the most powerful endopeptidase, and Cath B, which possesses endopeptidase and exopeptidase activity, Cath H acts mainly as exo(amino) peptidase (Koga et al, 1992; Kirschke et al, 1995). The linkage between the enzymatic properties of cysteine peptidases and their differential function in human lung as well as in cancer progression is not known. With the ELISA technique, we determined not only mature Cath H but also its proform and complexed Cath H molecules. The active enzyme status of Cath H in lung tissues and lung tumours might provide further information including correlation with lung diseases and cancer.

Since the difference in Cath H levels between non-tumourous lung parenchyma and tumours appeared in the group of smokers and not in the non-smokers, one may speculate that the level of Cath H expression is not mainly influenced by malignant progression but by the effects of cigarette smoking. A similar observation has been found for carcinoembryonic antigen (CEA) m-RNA levels in control and tumour lung tissues (Ohwada et al, 1995). On the other hand, extracellular levels of Cath H, being significantly increased and correlating with the stage of malignancy in breast, colorectal and melanoma cancers (Gabrijelčič et al, 1992; Kos et al, 1997), identify this enzyme as being active in tumour progression. Serum levels of Cath H determined in lung cancer patients clearly confirm the latter observations. Cath H levels were raised in sera of patients of all histological types, being the highest for SCLC and SCC. Patients suffering from benign lung diseases expressed lower levels of serum Cath H than patients with malignant tumours, but still significantly higher than those in healthy controls. Our results, revealing unchanged or decreased levels of Cath H in tumour tissues and increased levels in patients' sera, support the possibility that the secretion of Cath H is enhanced in lung tumours.

Lung tumours are very heterogeneous, expressing different metastatic abilities and then respond differently to chemo- and radiotherapy (Manegold and Drings, 1998; Schraube et al, 1998). Overall survival probability rate is very poor for patients with SCLC (Schiller, 1998). For patients with NSCLC the histological type and the tumour stage (pTNM) of the lung tumours are the most relevant prognostic indicators. However, within the groups of

 *© 2000 Cancer Research Campaign*

patients with low tumour stage, new prognostic factors are still needed to discriminate between high- and low-risk patients. Cath B has been shown to be a significant prognostic marker for NSCLC at enzyme activity (Ebert et al, 1994; Knoch et al, 1994; Werle et al, 1997*b*), and immunohistochemical levels (Inoue et al, 1994; Sukoh et al, 1994). Cath L and specific cysteine peptidase inhibitor stefin B have also been shown to be prognostic factors in lung cancer patients (Ebert et al, 1997; Werle et al, 1997*a*). In the most recent study Cath B immunostaining was demonstrated as a strong, independent prognostic factor in patients with SCC (Werle et al, 1999a).

For Cath H we found no significant correlation between tumour levels and survival when in the entire patient population was analysed. However, in the subgroup of smokers, Cath H levels correlated with the survival probability rate. Thus, cigarette smoking and high level of tumour Cath H may predict high risk of death in lung cancer patients.

In conclusion, in lung tumours Cath H shows a different behaviour from other cysteine peptidases, e.g. Cath B or Cath L. Its levels were lower in tumours but significantly higher in sera of lung cancer patients, suggesting either its lower expression or enhanced secretion or both. The prognostic impact of Cath H concentration in lung cancer is rather low and this is in agreement with the lack of correlation with the clinical and pathological parameters that indicate progression of lung cancer. However, the association of Cath H and cigarette smoking revealed a significantly increased risk of death in lung cancer patients and indicates the involvement of Cath H in tobacco-induced carcinogenesis.

### ACKNOWLEDGEMENTS

This work was supported by grants J3-6208 and RA 2320 (JK) from The Ministry of Science and Technology of the Republic of Slovenia and by Deutsche Krebshilfe-Dr. Mildred-Scheel-Stiftung 10-1085-Eb-1 (WE, ES). The authors thank Prof. Roger Pain for his critical reading of the manuscript and Beate Schaufler for skilful technical assistance.

### REFERENCES

Abel U, Berger J and Wiebelt H (1984) An exploratory procedure for the evaluation of quantitative prognostic factors. *Methods Inf Med* **23**: 154–156

Bradford MM (1976) A rapid and sensitive method for the quantitation of microgram quantities of protein utilizing the principle of protein dye-binding. *Anal Biochem* **72**: 248–254

Budihna M, Strojan P, Šmid L, Škrk J, Vrhovec J, Župevc A, Rudolf Z, Žargi M, Krašovec M, Svetic B, Kopitar-Jerala N and Kos J (1996) Prognostic value of cathepsins B, H, L, D and their endogenous inhibitors stefins A and B in head and neck carcinoma. *Biol Chem Hoppe-Seyler* **377**: 385–390

Chang JC, Lesser M, Yoo OH and Orlowski M (1986) Increased cathepsin B-like activity in alveolar macrophages and bronchoalveolar lavage fluid from smokers. *Am Rev Respir Dis* **134**: 538–541

Chapman HA, Riese RJ and Shi G-P (1997) Emerging roles for cysteine proteases in human biology. *Annu Rev Physiol* **59**: 63–88

Duffy MJ (1996) Proteases as prognostic markers in cancer. *Clin Cancer Res* **2**: 613–618

Ebert W, Knoch H, Werle B, Trefz G, Muley TH and Spiess E (1994) Prognostic value of increased lung tumor tissue cathepsin B. *Anticancer Res* **14**: 895–900

Ebert E, Werle B, Julke B, Kopitar-Jerala N, Kos J, Lah T, Abrahamson M, Spiess E and Ebert W (1997) Expression of cysteine protease inhibitors stefin A, stefin B, and cystatin C in human lung tumor tissue. *Adv Exp Med Biol* **421**: 259–265

Gabrijelčič D, Svetic B., Spaič D, Škrk J, Budihna M, Dolenc I, Popovič T, Cotič V and Turk V (1992) Cathepsins B, H and L in human breast carcinoma. *Eur J Clin Chem Clin Biochem* **30**: 69–74

Gairola CG, Galicki NI, Cardozo C, Lai YL and Lesser M (1989) Cigarette smoke stimulates cathepsin B activity in alveolar macrophages of rats. *J Lab Clin Med* **114**: 419–425

Hermenek P and Sobin L (1987) *UICC TNM classification of malignant tumors*, 4th edn. Springer Verlag: Berlin

Inoue T, Ishida T, Sugio K and Sugimachi K (1994) Cathepsin B expression and laminin degradation as factors influencing prognosis of surgically treated patients with lung adenocarcinoma. *Cancer Res* **54**: 6133–6136

Ishii Y, Hashizume Y, Kominami E and Uchiyama Y (1991*a*) Changes in immunoreactivity for cathepsin H in rat type II alveolar epithelial cells and its proteolytic activity in bronchoalveolar lavage fluid over 24 hours. *Anat Rec* **230**: 519–523

Ishii Y, Hashizume Y, Watanabe T, Waguri S, Sato N, Yamamoto M, Hasegawa S, Kominami E and Uchiyama Y (1991*b*) Cysteine proteinases in bronchoalveolar epithelial cells and lavage fluid of rat lung. *J Histochem Cytochem* **39**: 461–468

Kaplan EL and Meier P (1958) Non-parametric estimation from incomplete observation. *J Am Stat Assoc* **53**: 457–481

Kirschke H, Barrett AJ and Rawlings BJ (1995) Proteinases 1: lysosomal cysteine proteinases. In: *Protein Profile*, Sheterline P (ed), pp. 1584–1620. Academic Press: London

Knoch H, Werle B, Ebert W and Spiess E (1994) Imbalance between cathepsin B and cysteine proteinase inhibitors is of prognostic significance in human lung cancer. *Int J Oncol* **5**: 77–85

Koga H, Mori N, Yamada H, Nishimura Y, Tokuda K, Kato K and Imoto T (1992) Endo-and aminopeptidase activities of rat cathepsin H. *Chem Pharm Bull* **40**: 965–970

Kos J and Lah TT (1998) Cysteine proteinases and their endogenous inhibitors: target proteins for prognosis, diagnosis and therapy in cancer (review). *Oncol Rep* **5**: 1349–1361

Kos J, Šmid A, Krašovec M, Svetic B, Lenarčič B, Vrhovec I, Škrk J and Turk V (1995) Lysosomal proteinases cathepsins D, B, H, L and their inhibitors stefins A and B in head and neck cancer. *Biol Chem Hoppe-Seyler* **376**: 401–405

Kos J, Štabuc B, Schweiger A, Krašovec M, Cimerman N, Kopitar-Jerala N and Vrhovec I (1997) Cathepsins B, H, and L and their inhibitors stefin A and cystatin C in sera of melanoma patients. *Clin Cancer Res* **3**: 1815–1822

Krepela E, Kasafirek E, Novak K and Viklicky J (1990) Increased cathepsin B activity in human lung tumors. *Neoplasma* **37**: 61–70

Ledakis P, Tester WT, Rosenberg N, Romero-Fischmann D, Daskal I and Lah TT (1996) Cathepsins D, B, and L in malignant human lung tissue. *Clin Cancer Res* **2**: 561–568

Lesser M, Galicki N, Cardozo C and Gairola CG (1989) Cathepsin L activity in alveolar macrophages of rats: response to cigarette smoke. *Am J Respir Cell Mol Biol* **1**: 371–376

Manegold C and Drings P (1998) Chemotherapie des nichtkleinzelligen Lungenkarzinoms. In: *Thoraxtumoren: Diagnostik – Staging – gegenwärtiges Therapiekonzept*, Drings P and Vogt-Moykpf I (eds), pp. 310–327. Springer Verlag: Heidelberg

McCaughan BC, Martini N and Bains MS (1985) Bronchial carcinoids: review of 124 cases. *J Thorac Cardiovasc Surg* **89**: 8–17

Mountain CF (1991) Surgical treatment of lung cancer. *Critic Rev Oncol/Hematol* **11**: 179–207

Ohwada A, Takahshi H, Nagaoka I, Iwabuchi K, Mikami K and Kira S (1995) Effect of cigarette smoke on the mRNA and protein expression of carcinoembryonic antigen (CEA), a possible chemoattractant for neutrophils in human bronchioloalveolar tissues. *Thorax* **50**: 651–657

Qian F, Chan SJ, Gong Q, Bajkowski AS, Steiner DF and Frankfater A (1991) The expression of cathepsin B and other lysosomal proteinases in normal tissues and in tumors. *Biomed Biochim Acta* **50**: 531–540

San Segundo B, Chan SJ and Steiner DF (1986) Differences in cathepsin B mRNA levels in rat tissues suggest specialized functions. *FEBS Lett* **201**: 251–256

Schiller JH (1998) Lung cancer: therapeutic modalities and cytoprotection. *Lung* **176**: 145–164

Schmitt M, Jänicke F and Graeff H (1992) Tumor-associated proteases. *Fibrinolysis* **6**: 3–26

Schraube P, Kimmig B, Latz D, Flentje M and Wannenmacher M (1998) Radiotherapie des Bronchialkarzinoms. In: *Thoraxtumoren: Diagnostik – Staging – gegenwärtiges Therapiekonzept*, Drings P and Vogt-Moykopf I (eds), pp. 277–295. Springer Verlag: Heidelberg

Schweiger A, Štabuc B, Popovič T, Turk V and Kos J (1997) Enzyme-linked immunosorbent assay for the detection of total cathepsin H in human tissue cytosols and sera. *J Immunol Methods* **201**: 165–172

*© 2000 Cancer Research Campaign*

Sivaparvathi M, Sawaya R, Gokaslan ZL, Chintala KS and Rao JS (1996) Expression and the role of cathepsin H in human glioma progression and invasion. *Cancer Lett* **104**: 121–126

Sloane BF, Moin K and Lah TT (1994) Regulation of lysosomal endopeptidases in malignant neoplasia. In: *Biochemical and Molecular Aspects of Selected Cancers*, Pretlow TG and Pretlow TP (eds), pp. 411–472. Academic Press: New York

Sukoh N, Shosaku A, Ogura S, Isobe H, Takekawa H, Inoue K and Kawakami Y (1994) Immunohistochemical study of cathepsin B. Prognostic significance in human lung cancer. *Cancer* **74**: 46–51

Takahashi H, Ishidoh K, Muno D, Ohwada A, Nukiwa T, Kominami E and Kira S (1993) Cathepsin L activity is increased in alveolar macrophages and bronchoalveolar lavage fluid of smokers. *Am Rev Respir Dis* **147**: 1562–1568

Treftz G, Lüthgens K, Erdel M, Spiess E and Ebert W (1989) Plasminogen activator and cathepsin B in normal and malignant human lung tissue. *J Cancer Res Clin Oncol* **115**: S50

Uchiyama Y, Waguri S, Sato N, Watanabe T, Ishido K and Kominami E (1994) Review: cell and tissue distribution of lysosomal cysteine proteinases, cathepsins B, H and L, and their biological roles. *Acta Histochem Cytochem* **27**: 287–308

Werle B, Ebert W, Klein W and Spiess E (1995) Assessment of cathepsin L activity by use of the inhibitor CA-074 compared to cathepsin B activity in human lung tumor tissue. *Biol Chem Hoppe-Seyler* **376**: 157–164

Werle B, Jülke B, Lah T, Kos J, Spiess E and Ebert W (1997*a*) Cathepsin B and Cathepsin L: prognostic factors in human lung cancer? In: *Proteolysis in Cell Function*, Hopsu-Havu VK, Jarvinen M and Kirschke H (eds), pp. 472–478. IOS Press: Amsterdam

Werle B, Jülke B, Lah T, Spiess E and Ebert W (1997*b*) Cathepsin B fraction active at physiological pH of 7.5 is of prognostic significance in squamous cell carcinoma of human lung. *Br J Cancer* **75**: 1137–1143

Werle B, Lötterle H, Schanzenbächer U, Lah TT, Kalman E, Kayser K, Bülzebruck H, Schirren S, Krasovec M, Kos J and Spiess E (1999*a*) Immunochemical analysis of cathepsin B in lung tumours: an independent prognostic factor for squamous cell carcinoma patients. *Br J Cancer* **81**: 510–519

Werle B, Staib A, Jülke B, Ebert W, Zladoidsky P, Sekirnik A, Kos J and Spiess E (1999*b*) Fluorometric microassays for the determination of cathepsin L and cathepsin S activities in tissue extracts. *Biol Chem* **380**: 1109–1116

WHO (1981) *Histological Classification of Lung Tumours*. World Health Organization: Geneva

*© 2000 Cancer Research Campaign*
